# Comparison of six methods to estimate adherence in an ART-naïve cohort in a resource-poor setting: which best predicts virological and resistance outcomes?

**DOI:** 10.1186/s12981-017-0138-y

**Published:** 2017-04-04

**Authors:** Catherine Orrell, Karen Cohen, Rory Leisegang, David R. Bangsberg, Robin Wood, Gary Maartens

**Affiliations:** 1 Department of Medicine, Desmond Tutu HIV Centre, Institute of Infectious Disease and Molecular Medicine, University of Cape Town, Cape Town, South Africa; 2 Division of Clinical Pharmacology, Department of Medicine, University of Cape Town, Cape Town, South Africa; 3 Harvard Medical School, Boston, MA USA; 4 Massachusetts General Hospital Center for Global Health, Boston, MA USA; 5 Ragon Institute of Massachusetts General Hospital, Boston, MA USA; 6 Desmond Tutu HIV Centre, Werner Beit Building North, UCT Faculty of Health Science, Anzio Road, Observatory, Cape Town, 7925 South Africa

**Keywords:** HIV, Antiretroviral therapy, Adherence, Electronic monitoring, Virological outcome, HIV-1 resistance, Genotyping

## Abstract

**Background:**

Incomplete adherence to antiretroviral therapy (ART) results in virologic failure and resistance. It remains unclear which adherence measure best predicts these outcomes. We compared six patient-reported and objective adherence measures in one ART-naïve cohort in South Africa.

**Methods:**

We recruited 230 participants from a community ART clinic and prospectively collected demographic data, CD4 count and HIV-RNA at weeks 0, 16 and 48. We quantified adherence using 3-day self-report (SR), clinic-based pill count (CPC), average adherence by pharmacy refill (PR-average), calculation of medication-free days (PR-gaps), efavirenz therapeutic drug monitoring (TDM) and an electronic adherence monitoring device (EAMD). Associations between adherence measures and virologic and genotypic outcomes were modelled using logistic regression, with the area under the curve (AUC) from the receiver operator characteristic (ROC) analyses derived to assess performance of adherence measures in predicting outcomes.

**Results:**

At week 48 median (IQR) adherence was: SR 100% (100–100), CPC 100% (95–107), PR-average 103% (95–105), PR-gaps 100% (95–100) and EAMD 86% (59–94), and efavirenz concentrations were therapeutic (>1 mg/L) in 92%. EAMD, PR-average, PR-gaps and CPC best predicted virological outcome at week 48 with AUC ROC of 0.73 (95% CI 0.61–0.83), 0.73 (95% CI 0.61–0.85), 0.72 (95% CI 0.59–0.84) and 0.64 (95% CI 0.52–0.76) respectively. EAMD, PR-gaps and PR-average were highly predictive of detection of resistance mutations at week 48, with AUC ROC of 0.92 (95% CI 0.87–0.97), 0.86 (0.67–1.0) and 0.83 (95% CI 0.65–1.0) respectively. SR and TDM were poorly predictive of outcomes at week 48.

**Conclusion:**

EAMD and both PR measures predicted resistance and virological failure similarly. Pharmacy refill data is a pragmatic adherence measure in resource-limited settings where electronic monitoring is unavailable.

*Trial registration* The trial was retrospectively registered in the Pan African Clinical Trials Registry, number PACTR201311000641402, on the 13 Sep 2013 (www.pactr.org). The first participant was enrolled on the 12th July 2012. The last patient last visit (week 48) was 15 April 2014

**Electronic supplementary material:**

The online version of this article (doi:10.1186/s12981-017-0138-y) contains supplementary material, which is available to authorized users.

## Background

Adherence is critical to realising the clinical and prevention benefits of ART [1–3]. Despite this, there is no gold standard identifying adherence levels and patterns that place individuals at risk for virologic failure and/or drug resistance [4–6].

A variety of ART adherence measures have been used in both observational and intervention studies. Self-report is the most frequently used method, but is often imprecise and overestimates adherence, similar to clinic-based pill count [4–7]. Pharmacy refill data also overestimate adherence, but have been consistently associated with virological failure and mortality [8–10]. Electronic drug monitoring methods have been closely associated with virologic failure, despite underestimating adherence due to storage and ingestion of medications outside of the device (pocket-doses) [6, 11, 12]. Therapeutic drug concentration monitoring has been associated with virologic outcomes, though its precision relative to other adherence monitoring approaches is poorly understood [3, 13, 14].

Few studies have directly compared the ability of multiple adherence monitoring methods to predict virological failure in one cohort [15–17] or explored their relationship with drug resistance [18].

Our study compared a variety of adherence measures, three widely accessible to clinicians in a resource-poor setting: self-report (SR), clinic-based pill count (CPC) and pharmacy refill data (PR-average); and three requiring additional technology or calculation: therapeutic drug monitoring (TDM), calculation of medication free days or gaps (PR-gaps) and real-time electronic drug monitoring. All adherence measures were collected in a prospective ART-naïve cohort starting first-line ART, in order to explore which measure best predicted virological or resistance outcome at 16 and 48 weeks into treatment.

## Methods

### Participants and setting

This study is a sub-study of a randomised controlled trial recruited at the Hannan Crusaid Treatment Centre, an outpatient antiretroviral treatment centre in Cape Town, South Africa. This site and the study have been described elsewhere [19, 20]. The cohort included ART-naïve adults and adolescents (≥12 years) starting first-line ART. Participants were eligible for the parent study if they had their own mobile phone, signed an informed consent, and had either a baseline CD4 count below 350 cells/µl or a stage 3 or 4 AIDs-defining illness in keeping with the national HIV guidelines for starting ART [20]. All patients on the parent study with viral load data available at week 16 or week 48 were included in this sub-study.

### Standard of care (SoC)

All participants received three group counsellor-driven treatment preparedness sessions prior to commencing ART [21, 22]. They were visited at home by a community care worker to confirm address and ascertain home circumstances. All participants commenced a non-nucleoside reverse transcriptase inhibitor (NNRTI)-based first-line ART regimen. SoC visit frequency and procedures are detailed in Additional file 1: Table S1. *Counts of pill returns* were done at each scheduled clinic visit. Those with a raised HIV-RNA (>1000 copies/ml) or non-optimal clinic-based pill count adherence (<90%) received additional adherence support, which included tailored counselling, monthly drug dispensing and follow-up home-visits. Participants were traced by phone and home visit if they were more than 4 weeks late in attending a scheduled clinic visit.

### Study design and procedures

The parent study was a randomised controlled trial, with primary outcome at 48 weeks, investigating the impact of mobile phone message reminders when missed doses were detected by a real-time electronic adherence monitoring device (EAMD) on adherence to ART [20]. This device has been used in other resource-limited settings [23, 24]. This sub-study includes data from participants without reference to study arm.

There were five study visits: screening (week-4), baseline (week 0), weeks 16, 32 and 48, detailed in Table 1. Participants were reimbursed for local travel (~US$2) at each visit and for the three on-study visits (weeks 16, 32 and 48) were given a gift of a T-shirt, bag or mug valued at R80 (~US$8) or less. If participants came to a SoC clinic visit but did not attend the corresponding study visit, tablet return and virological data were extracted from their clinic folder.

**Table 1:** Baseline demographic, clinical treatment and psychosocial characteristics of participants

Variable	Full cohort	With VL at week 16	With VL at week 48
Number: *n*	230	160	180
Female sex: *n* (%)	150 (65.2)	108 (67.5)	121 (67.2)
Age in years: mean (SD)	34.5 (9.1)	34.8 (8.9)	35.0 (9.4)
Height (cm): mean (SD)	164.0 (8.6)	164.1 (8.2)	164.0 (8.1)
Weight (kg): median (IQR)	67.3 (57.8–79.6)	67.2 (58.0–80.0)	68.1 (58.7–80.4)
BMI: median (IQR)	24.3 (21.3–29.8)	24.2 (21.5–29.9)	24.6 (21.5–30.7)
WHO stage: *n* (%)			
1	84 (36.5)	58 (36.3)	73 (40.6)
2	47 (20.4)	34 (21.3)	39 (21.7)
3	75 (32.6)	54 (33.8)	51 (28.3)
4	24 (10.4)	14 (8.8)	17 (9.4)
CD4 count (cells/mm^3^): median (IQR)	225 (133–287)	229 (132–288)	233 (144–287)
Log HIV-RNA (copies/ml): median (IQR)	4.9 (4.4–5.4)	4.9 (4.4–5.4)	4.8 (4.4–5.4)
NNRTI at start: *n* (%)			
Efavirenz	228 (99.1)	(98)	
Nevirapine	2 (0.9)	2 (1.2)	2 (1.1)
NRTI at start^a^: *n* (%)			
Tenofovir	225 (97.8)	159 (99.4)	177 (98.3)
Zidovudine	4 (1.7)	1 (0.6)	3 (1.7)
Stavudine	1 (0.4)	–	–
HADS depression score of 8 or above (borderline or case)^b^: *n* (%)	74 (32.1)	55 (34.3)	58 (32.2)
HADS anxiety score of 8 or above (borderline or case)^b^: *n* (%)	89 (38.7)	64 (40.0)	70 (38.9)
Non-disclosure: *n* (%)	11 (4.7)	8 (5.0)	7 (3.9)
CAGE score ≥ 2: *n* (%)	35 (15.2)	22 (13.8)	25 (13.9)

^a^All were taking 3TC or FTC as a second NRTI

^b^14-question Hospital Anxiety and Depression Score

Demographic and psychosocial details, including age, gender, weight, height as well as assessments for depression, anxiety and alcohol use, were collected at screening. The 14-question Hospital Anxiety and Depression Score (HADS) was used to assess anxiety and depression and the CAGE score used to assess alcoholism [25, 26]. Blood was drawn for CD4 cell count (FACS Count™, Beckton Dickinson, NJ, USA) and HIV-RNA (HIV-1 RNA 3.0 assay^®^, Bayer Healthcare, Leverkusen, Germany) at baseline, 16 week and 48 week visits. Mid-dose efavirenz concentrations were determined at weeks 16 and 48. The time of blood draw, and the self-reported time of most recent ART dosing were recorded.

### Adherence data

For pill counts, average pharmacy refill data and self-recall, the data used was that typically available to clinic staff during a consultation with a patient.

### Three-day self-recall (SR)

At weeks 16 and 48, study staff asked each participant: “Did you swallow your pills yesterday/2 days ago/3 days ago?” Percent doses taken over 3 days was calculated from the participants’ responses. Study staff were not part of the clinical team. Data were only available if a participant attended the study visit.

#### Clinic-based pill count (CPC)

Participants were instructed to bring any remaining tablets to each study visit. CPC adherence was calculated using the difference between the number of tablets dispensed and the number returned and dividing by the number of days between the date of dispensing and the current visit date (adjusted for the number of doses per day). At week 16 this would give adherence over the previous 1 month period (last dispensing visit was the SoC visit a week 12) and at week 48 adherence over the previous 2 month period (last dispensing visit was the SoC visit at week 40). For the analyses, only the tablet count related to dosing efavirenz, nevirapine or, for those who switched to second-line, lopinavir/r dosing was used.

#### Pharmacy refill: average method (PR-average)

An electronic dispensing system (iDART) was used at the site to record the date of ART dispensed and the quantity given to each participant [27, 28]. Obvious errors, such as date and dispensing duplications were removed. A cumulative PR-average measure was obtained at week 16 and week 48 visits by dividing the number of days of efavirenz, nevirapine or lopinavir/r tablets each patient received between study randomisation date and the visit date, by the number of days they were in care over the same period. This method averages adherence across the whole period in question.

#### Pharmacy refill: gaps method (PR-gaps)

This measure used pharmacy dispensing quantities and refill visit dates to determine the number of medication-free days (days when the participant could not have had medication in hand) in each dispensing period. The number of medication-free days are subtracted from the number of days in the period, and the result divided by the number of days in the period (up to week 16 or up to week 48) to give an adherence percentage [10]. This method identifies days when it was not possible for a patient to have taken medication; and might be expected to yield lower but potentially more accurate median adherence values than the average method.

#### Therapeutic drug measuring (TDM)

At weeks 16 and 48 visits a sample was taken to quantify mid-dosing interval efavirenz concentrations. Mid-dosing interval samples were drawn in the morning, after dosing the previous evening. Self-reported time of the evening dose was recorded. Samples were kept cold (4 °C) until transfer to the laboratory within 2–3 h of blood draw. Samples were centrifuged at 3500 revolutions/min for 10 min and plasma pipetted into cryovials which were labelled and frozen at −80 °C. Efavirenz concentrations were analysed using a validated LC–MS/MS method. The therapeutic range for mid-dose EFV concentrations is 1–4 mg/L.

#### EAMD data

All participants received the EAMD on study entry to record daily ART dosing times. The device (Wisepill^®^) held a week of medication in an internal and removable seven compartment pill box container. Participants were trained to fill these pill boxes themselves and replace them once a week at the same time as they dosed. On opening, a signal was sent via the mobile-phone network to a secure central computer, thus recording tablet taking or treatment interruptions in real time. An adherent day was defined as any recorded EAMD opening from 06h00am on that day to 05h59am the following day. Days with battery voltage <3660 V or no heartbeat were censored (dead battery days). The daily EAMD data for each participant were downloaded from the Wisepill^®^ server. Cumulative adherence data for EAMD was calculated as the number of adherent days divided by the number of days in care, from date of randomisation to either weeks 16 or 48.

### Study outcomes

#### Virological failure

Failure was defined as a single HIV RNA of >400 copies/ml at week 16 or >40 copies/ml at week 48 [29, 30].

#### HIV-1 drug resistance

Genotype resistance tests were done on those with HIV RNA >500 copies/ml (the minimum HIV-RNA for amplification) at weeks 16 or 48. Nucleic acid was extracted with the NucliSens EasyMag automated extraction system (bioMérieux, Boxtel, The Netherlands). Genotyping was performed by RT-PCR followed by a nested PCR and sequencing of the complete PR gene and RT codons 1–262 (HXB2 numbering: HIV-1 nucleotide positions 2250–4229) and using gene-specific sequencing primers [31]. Participants with one or more major resistance mutation as defined by the 2014 IAS update of drug resistance in HIV-1, causing resistance to ≥1 of the three drugs in their regimen were classified as resistant [32].

### Statistical analysis

Data were analysed using Stata 13.0 (Stata Corporation, College Station, USA). Descriptive statistics (number, percentage, median and interquartile ranges) were used to summarise the baseline characteristics of the participant group and to tabulate the adherence data.

All available adherence data were used from each individual who had a HIV-RNA drawn within a 4 week window around week 16 (weeks 12–20) or a 16 week window around week 48 (weeks 32–64) in a per-protocol analysis from the time they entered care until the date of the respective viral load.

Univariate and multivariable logistic regression models were used to explore each adherence measure’s relationship to virological failure and genotypic resistance at weeks 16 and 48. Both outcomes were binary, categorised as described above. The data for each adherence variable was continuous, although models were repeated using categorical variables (binary or quartiles) to assess linearity. For the TDM models, the log_10_ values of EFV mid-dose drug concentrations were used, as log_10_ values shift the distribution curve of the EFV concentration values toward normal for regression modelling.

Other variables included in the models (age, baseline CD4 cell count and baseline HIV-RNA) were specified prior to the analysis. Receiver Operator Characteristics (ROC) were generated to view the overall predictive power of the univariate and multivariable models. The area under the curve derived from the ROC (AUC ROC), with 95% confidence intervals, was used to compare the prediction of virological failure or resistance by each adherence measure. AUC ROCs were compared using non-parametric methods.

## Results

**Fig. 1:**
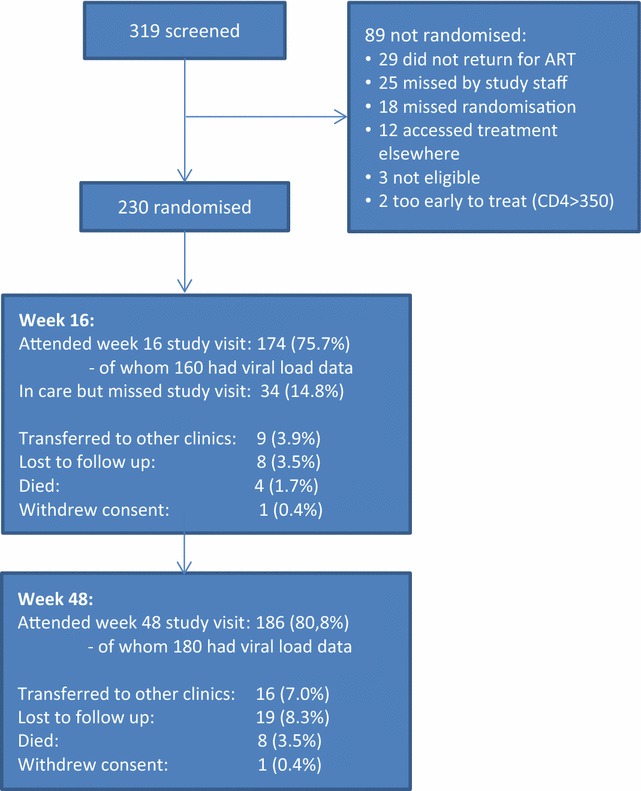
Flow diagram describing the outcome of the 319 individuals screened and the 230 individuals randomised to the study

Two-hundred-thirty antiretroviral naïve HIV-positive participants were recruited onto the study between 09 July 2012 and 09 April 2013. Figure 1 is a flow diagram of study participants. Baseline characteristics are described in Table 1. The cohort is typical of other African ART cohorts, with a predominance of women. The majority were clinically well. More than a third of the cohort had anxiety or depression scores (>8) that required further assessment and 15% were alcoholic on CAGE (score of ≥2). These data did not significantly impact on adherence in the parent study [20]. A minority had not disclosed their HIV-status to any other person.

### Antiretroviral therapy

Efavirenz-based ART was initiated in 228 participants and the remaining two started twice-daily nevirapine-based ART. Both regimens were given with two background nucleoside reverse transcriptase inhibitors. Tenofovir/3TC were used in 97.8% (Table 1). The majority of participants were taking three tablets once a day during the study period.

### Retention in care (Fig. 1)

At the end of the study 186 participants remained in care (80.8%) at the study site and a further 16 (7.0%) had transferred to care elsewhere. Eight participants (3.5%) had died and 19 (8.3%) were lost to follow up i.e. had not attended the clinic for more than 12 weeks and were not known to have transferred out or to have died. One participant withdrew consent.

### Adherence measures

Table 2 describes the median adherence by each measure at weeks 16 and 48, for all participants with a HIV-RNA drawn at that visit. Three-day self-recall (SR) and efavirenz therapeutic drug monitoring (TDM) data were only available if study visits were completed. Clinic-based pill count (CPC) and pharmacy refill data (PR-gaps and PR-average) were available for all those who attended the clinic. EAMD data were available for all study participants. There were 82,311 participant dosing days recorded, and of these, 8362 (10.1%) were dead battery days.

**Table 2:** Median adherence percentage with inter-quartile range at each study visit, by adherence measure

	*n*	Week 16	*n*	Week 48
Self-report (%): median (IQR)	140	100 (100–100)	169	100 (100–100)
Pill count (%): median (IQR)	160	100 (92–100)	178	100 (95–107)
Average pharmacy refill (%): median (IQR)^a^	158	104 (101–105)	178	103 (95–105)
Gaps pharmacy refill (%): median(IQR)^a^	158	100 (100–100)	178	100 (95–100)
EAMD adherence (%): median (IQR)^a^	160	93 (74–98)	180	86 (59–94)
EFV concentration (mg/L): median(IQR)	136	2.3 (1.6–4.4)	156	2.1 (1.5–3.4)
Viral suppression: *n* (%)	160	146 (91.2)	180	153 (85.0)

Self-report and EFV concentrations are measured on the date of the visit. Tablet counts cover the 30 days (week 16) or 60 days (week 48) before the visit. Pharmacy refill and EAMD adherence are cumulative data from baseline to latest time in care

^a^Cumulative per protocol measures

The subjective measure, SR, gave the highest adherence with the most individuals reporting 100% adherence (median 100%, IQR 100–100%). CPC, PR-gaps and PR-average also gave perfect or close to perfect adherence at week 16 and 48, but widened inter-quartile ranges highlighted some variations within the participants. Cumulative adherence by EAMD showed the lowest median adherence at both visits: 93% (IQR 74–98%) at week 16 and 86% (IQR 59–94%) at week 48. Median efavirenz concentrations were 2.3 (IQR 1.6–4.4) mg/L at week 16, and 2.1 (IQR 1.5–3.4) mg/L at week 48, within the currently recognised therapeutic range of 1.0–4.0 mg/L at both time points.

### Virological outcome

At week 16, 160 participants had HIV-RNA available within the 12–20 week window and 146 (91.2%) were suppressed to ≤400 copies/ml (Table 1). At week 48, 180 had a HIV-RNA available, of whom 153 (85.0%) were suppressed to ≤40 copies/ml (Table 1). Three participants switched to second-line therapy between weeks 32 and 48 of the study.

### Virological failure prediction models

At week 48, adherence measured by EAMD, PR-average, PR-gaps and CPC significantly predicted virological failure both in univariate and multivariable analyses. The odds ratio for these models gives the reduction in the risk of failure for each 10% increase in adherence as quantified by the specified method (Table 3). Neither TDM nor SR adherence predicted failure. For all multivariable models, an increased CD4 cell count at baseline was associated with reduced odds of failure (continuous model examples are presented in Additional file 1: Table S2; and categorical model examples in Additional file 1: Table S3).

**Table 3:** The table below presents odds ratios (OR) or adjusted odds ratios (aOR) with 95% confidence intervals (CI) for failure or resistance given a 10% increase in the adherence variable (or a 1 log increase in EFV concentration) in each logistic regression model

Adherence measure	Univariate model		Multivariable model	
	OR (95% CI)	p value	aOR (95% CI)	p value
Virological failure (>40 copies/ml) at week 48				
EAMD^a^	0.87 (0.82–0.94)	*<0.001*	0.89 (0.82–0.95)	*0.001*
PR-average	0.78 (0.70–0.88)	*<0.001*	0.78 (0.69–0.87)	*<0.001*
PR-gaps	0.69 (0.56–0.82)	*<0.001*	0.68 (0.56–0.82)	*<0.001*
CPC	0.89 (0.82–0.96)	*0.004*	0.88 (0.80–0.96)	*0.004*
Log_10_EFV	0.40 (0.16–1.03)	0.059	0.52 (0.19–1.37)	0.184
SR	0.98 (0.89–1.08)	0.698	0.98 (0.88–1.09)	0.720
Resistance (presence of ≥1 major mutation) at week 48				
EAMD	0.74 (0.64–0.87)	*<0.001*	0.68 (0.53–0.88)	*0.003*
PR-average	0.77 (0.69–0.87)	*<0.001*	0.77 (0.66–0.89)	*<0.001*
PR-gaps	0.74 (0.65–0.85)	*<0.001*	0.77 (0.66–0.89)	*0.001*
CPC	0.85 (0.77–0.94)	*0.002*	0.82 (0.69–0.98)	*0.031*
Log_10_EFV	0.14 (0.04–0.45)	*0.001*	0.14 (0.02–0.78)	*0.025*
SR	0.92 (0.83–1.02)	0.102	0.92 (0.78–1.08)	0.316
Virological failure (>400 copies/ml) at week 16				
EAMD	0.93 (0.86–1.01)	0.085	0.96 (0.87–1.06)	0.459
PR-average	0.68 (0.55–0.83)	*<0.001*	0.66 (0.50–0.73)	*0.004*
PR-gaps	0.64 (0.51–0.82)	*<0.001*	0.64 (0.47–0.88)	*0.006*
CPC	0.89 (0.78–1.03)	0.133	0.94 (0.78–1.13)	0.491
Log_10_EFV	0.17 (0.04–0.75)	*0.020*	0.20 (0.03–1.47)	0.115
SR	0.92 (0.83–1.03)	0.163	0.98 (0.83–1.01)	0.765
Resistance (presence of ≥1 major mutation) at week 16				
EAMD	0.93 (0.85–1.01)	0.085	0.96 (0.86–1.07)	0.435
PR-average	0.85 (0.72–0.99)	*0.036*	0.87 (0.72–1.06)	0.171
PR-gaps	0.85 (0.71–1.01)	0.066	0.88 (0.69–1.11)	0.287
CPC	0.90 (0.80–1.01)	0.069	0.93 (0.82–1.06)	0.316
Log_10_EFV	0.34 (0.08–1.81)	0.228	0.43 (0.07–2.66)	0.362
SR	0.93 (0.83–1.03)	0.147	0.97 (0.85–1.11)	0.647

Univariate models use only the adherence variable in the model with the outcome variable, multivariable models include the adherence variable and three baseline variables (CD4 cell count, log HIV-RNA and age) with the outcome variable. There are four outcome variables: the risk of virological failure to >40 copies/ml at week 48, the risk of virological failure to >400 copies/ml at week 16, the risk of resistance (presence of ≥1 IAS major mutation at genotyping) at weeks 16 and 48

^a^EAMD = electronic adherence monitoring device data; PR-average = average pharmacy refill data; PR-gaps = pharmacy refill gaps data; CPC = clinic-based pill count data; EFV = efavirenz mid-dosing interval data; SR = 3-day self-recall data

Those results that are significant (p < 0.05) are in italics

**Fig. 2:**
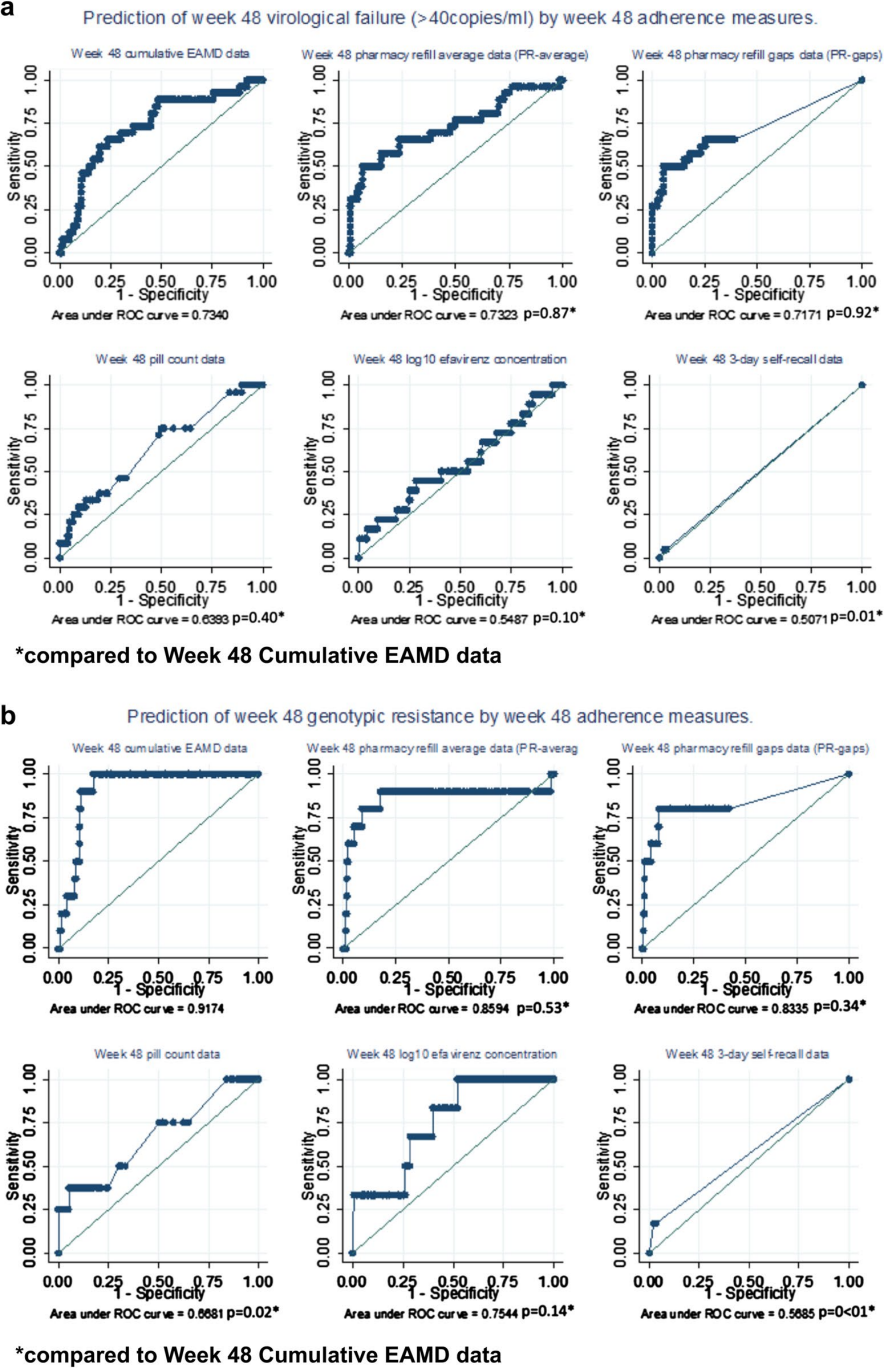
Prediction of virological failure or resistance by each adherence measure at week 48. Receiver operating characteristic (ROC) curves showing prediction of virological failure (>40 copies/ml) and resistance at week 48 by six adherence measures. Univariate model data are shown. **a** Prediction of virological failure to <40 copies/ml at week 48 using adherence measures at week 48. **b** Prediction of resistance at week 48 using adherence measures at week 48. *Compared to week 48 cumulative EAMD data

ROC curves were generated from the univariate models at 48 weeks, and AUC ROCs were derived to allow for comparison of predictive value across adherence methods (Fig. 2a). Cumulative measures EAMD (ROC AUC 0.73, 95% CI 0.61–0.83), PR-average (ROC AUC 0.73, 95% CI 0.61–0.85), PR-gaps (ROC AUC 0.72, 95% CI 0.59–0.84) and CPC (ROC AUC 0.64, 95% CI 0.52–0.76) best predicted failure at week 48.

At week 16, analyses showed that both PR-average and PR-gaps were predictive of failure, in both univariate and multivariable models. Log_10_ EFV concentration also reached significance in the univariate model (for every 10 times or 1 log increase in EFV concentration, the odds of failure reduced by 83%), but the other adherence measures, including EAMD, did not significantly predict failure at week 16 (Table 3).

### Resistance outcome

Twelve of the fourteen participants whose HIV-RNA was >400 copies/ml at week 16 were successfully amplified when sent for HIV-1 resistance genotyping. Two participants had no major resistance mutations. Eight (66.7%) participants expressed the K65R mutation-conferring resistance to tenofovir, and three the M184 V mutation- conferring resistance to lamivudine/emtricitabine. All 10 who expressed resistance had NNRTI mutations, including L100I, K101E, K103N, V106M, Y181C, Y188C/Y/L, G190A/G/S and H221HY. Despite only efavirenz and nevirapine being used, mutations conferring resistance to etravirine and rilpivirine were noted in three participants: one each of V90I, E138A and V179D.

Twenty-seven participants had a HIV RNA of >40 copies/ml at week 48. Eleven of the fourteen (51.9%) who had HIV RNA >500 copies/ml had resistance genotyping successfully completed. One had no major resistance mutations. Two (18%) participants expressed the K65R mutation and four (36%) the M184 V mutation. As at week 16, the majority of the remaining mutations, in all 11 who expressed resistance, were NNRTI mutations, including L100I, K101E, K103N, V106M, Y181C, G190A, H221HY and F227L. Again, mutations to etravirine and rilpivirine were noted in 3 participants: one with E138A and two with V179D.

### Resistance prediction models

As for virological failure, each adherence variable was modelled against the risk of resistance using both univariate and multivariate logistic regression models for prediction. Adherence as quantified by EAMD, PR-average, PR-gaps, TR and log_10_ EFV concentration were all significantly predictive of resistance in both univariate and multivariable analyses at week 48 (Table 3). SR was non-significant in either model. Reduced age and decreased CD4 cell count at baseline significantly increased the odds of resistance in all multivariable models (data not shown). AUC ROC values were obtained to allow for inter-measure comparison of resistance prediction at weeks 48 and 16. While cumulative EAMD best predicted resistance with the narrowest confidence intervals (AUC ROC 0.92, 95% CI 0.87–0.97), it was not significantly better than either PR measure (PR-average AUC ROC 0.86, 95% CI 0.67–1.0; PR-gaps AUC ROC 0.83, 95% CI 0.65–1.00) at week 48.

In contrast, only PR-average predicted resistance at week 16 (AUC ROC 0.72, 95% CI 0.57–0.90) (Table 3). Reduced age and decreased CD4 cell count at baseline significantly increased the odds of resistance in all multivariable models at week 16. All data on which theses analyses are based are available in Additional file 2.

## Discussion

We found high levels of adherence using both short-term and cumulative measures. Three-day SR yielded the highest adherence estimate, but was not a significant predictor of either viral suppression or resistance. This is consistent with findings which show that while short-term SR measures are widely utilised and useful for clinical intervention [33], they overestimate adherence, likely due to recall and social desirability bias [34–37], and are unreliable measures for research purposes.

In contrast, four of the five objective adherence measures effectively predicted virological failure: CPC, PR-average, PR-gaps and EAMD data. Cumulative PR measures (PR-average and PR-gap) were among the best predictors of virological failure and resistance at 48 and, of note, PR-average was the only predictor of resistance at week 16. PR-average has previously been reported to be a reliable predictor of virological outcomes and mortality [3, 8, 9], while our group demonstrated that short term PR-gap can predict failure on second-line ART [10]. Software to calculate PR-average or PR-gaps could easily be added to electronic dispensing systems, which are widely used in resource-limited settings. Programmatic review of adherence using PR methods could be implemented immediately.

CPC is not widely recommended as it is subject to “pill dumping” and can be complex to perform in a large clinic [3]. Nonetheless, CPC, which is a standard of care procedure in our clinic, performed well in this study and was predictive of virological failure and resistance at week 48.

While EAMD data were among the best at predicting outcome in this study at week 48, EAMDs are not routinely used in clinical care. However, recent data shows EAMD can reduce costs associated with HIV-RNA monitoring and real-time devices can detect early virologic rebound before established failure [3, 38, 39]. With the availability of newer, more affordable real-time technologies, electronic strategies should be reconsidered [40].

By altering the distribution of efavirenz concentrations through the use of log values in the regression model, we found that the log values of mid-dose EFV concentration were predictive of resistance at week 48, with wide confidence intervals, but not of failure. Most drug concentrations were therapeutic, possibly reflecting white coat pre-visit dosing, leaving few with concentrations below therapeutic where virological failure would be more likely. The few participants with sub-therapeutic concentrations limit the interpretation of this data. Larger studies including more participants with sub-therapeutic EFV concentrations are needed to adequately explore the ability of TDM to predict virological failure and resistance.

Most participants remaining in care at weeks 16 and 48 had virological suppression. The majority of those who had detectable HIV RNA also had resistance that would compromise at least one drug in their antiretroviral regimen, even at 16 weeks. Only the PR-average method predicted this early resistance. Using early pharmacy refill data to predict failure is practical and easy to achieve both for an individual and on a programmatic level. EAMD data was highly predictive of resistance at week 48, as were both PR measures and, to a lesser extent CPC.

It is interesting that while many of the adherence measures are predictive of viral or resistance outcomes at week 48, far fewer are predictive at week 16. This might suggest that measures of adherence which cover a longer period of dosing are more accurate e.g. cumulative EAMD, PR methods and CPC over 2-months. Conversely, adherence data collected over a shorter window were not predictive e.g. 3-day self-report, spot EFV concentrations and CPC over 1 month.

The prospective collection of multiple adherence measures in a single cohort is the strength of our study as it has allowed direct comparison of these measures—a comparison rarely achieved [13, 16]. Our study was based on maximum use of existing adherence data and used a per protocol approach to analysis, with those who did not have virological data at weeks 16 or 48 treated as missing for the predictive models. Losses to care in this cohort were similar to those previously reported at this site. All participants had data included in the cumulative adherence measures [41, 42].

Both genotyping and viral load testing are costly and funding to conduct baseline resistance testing and confirmatory viral loads at week 48 was not available. This is a study limitation. In addition, SR data was only collected as a 3-day recall at each visit and CPC data were only available for the 1 or 2 months preceding the week 16 or 48 visit and not over the complete study period. These measures may have performed better being collected over a longer or cumulative period of time.

## Conclusion

Adherence as measured by CPC, PR measures and EAMD were the best predictors of resistance and virological failure in this prospective study. Pill counting can be implemented at any clinic, and pharmacy refill data is already widely available and an immediately implementable option, particularly in resource-poor settings. Consideration should also be given to the use of electronic measures as adherence monitoring strategies as costs reduce [40].

## Additional files

**Additional file 1.** Supplementary tables.

**Additional file 2.** Study data on which manuscript analyses are based.

## References

[1] Paterson DL, Swindells S, Mohr J, Brester M, Vergis EN, Squier C (2000). Adherence to protease inhibitor therapy and outcomes in patients with HIV infection. Ann Intern Med.

[2] Bangsberg DR, Perry S, Charlebois ED, Clark RA, Roberston M, Zolopa AR (2001). Non-adherence to highly active antiretroviral therapy predicts progression to AIDS. AIDS.

[3] Thompson MA, Mugavero MJ, Amico KR, Cargill VA, Chang LW, Gross R (2012). Guidelines for improving entry into and retention in care and antiretroviral adherence for persons with HIV: evidence-based recommendations from an International Association of Physicians in AIDS Care panel. Ann Intern Med.

[4] Glass T, Cavassini M (2014). Asking about adherence—from flipping the coin to strong evidence. Swiss Med Wkly.

[5] Marcellin F, Spire B, Carrieri MP, Roux P (2013). Assessing adherence to antiretroviral therapy in randomized HIV clinical trials: a review of currently used methods. Expert Rev Anti Infect Ther.

[6] Gill CJ, Hamer DH, Simon JL, Thea DM, Sabin LL (2005). No room for complacency about adherence to antiretroviral therapy in sub-Saharan Africa. AIDS.

[7] Williams AB, Amico KR, Bova C, Womack JA (2013). A proposal for quality standards for measuring medication adherence in research. AIDS Behav.

[8] Bisson GP, Gross R, Bellamy S, Chittams J, Hislop M, Regensberg L (2008). Pharmacy refill adherence compared with CD4 count changes for monitoring HIV-infected adults on antiretroviral therapy. PLoS Med.

[9] Nachega JB, Hislop M, Dowdy DW, Lo M, Omer SB, Regensberg L (2006). Adherence to highly active antiretroviral therapy assessed by pharmacy claims predicts survival in HIV-infected South African adults. J Acquir Immune Defic Syndr.

[10] Court R, Leisegang R, Stewart A, Sunpath H, Murphy R, Winternheimer P (2014). Short term adherence tool predicts failure on second line protease inhibitor-based antiretroviral therapy: an observational cohort study. BMC Infect Dis.

[11] Marcellin F, Spire B, Carrieri MP, Roux P (2013). Assessing adherence to antiretroviral therapy in randomized HIV clinical trials: a review of currently used methods. Expert Rev Anti-Infect Ther.

[12] Haberer JE, Kiwanuka J, Nansera D, Muzoora C, Hunt PW, So J (2013). Realtime adherence monitoring of antiretroviral therapy among HIV-infected adults and children in rural Uganda. AIDS.

[13] Liu X, Ma Q, Zhang F (2010). Therapeutic drug monitoring in highly active antiretroviral therapy. Expert Opin Drug Saf.

[14] Kredo T, Van der Walt JS, Siegfried N, Cohen K. Therapeutic drug monitoring of antiretrovirals for people with HIV. Cochrane Database Syst Rev. 2009. (3):CD007268. doi:10.1002/14651858.CD007268.pub2. 10.1002/14651858.CD007268.pub2 19588422

[15] Liu H, Golin CE, Miller LG, Hays RD, Beck CK, Sanandaji S (2001). A comparison study of multiple measures of adherence to HIV protease inhibitors. Ann Intern Med.

[16] Oyugi JH, Byakika-Tusiime J, Charlebois ED, Kityo C, Mugerwa R, Mugyenyi P (2004). Multiple validated measures of adherence indicate high levels of adherence to generic HIV antiretroviral therapy in a resource-limited setting. J Acquir Immune Defic Syndr.

[17] Muller AD, Jaspan HB, Myer L, Hunter AL, Harling G, Bekker LG (2011). Standard measures are inadequate to monitor pediatric adherence in a resource-limited setting. AIDS Behav.

[18] Bangsberg DR, Hecht FM, Charlebois ED, Zolopa AR, Holodniy M, Sheiner L (2000). Adherence to protease inhibitors, HIV-1 viral load, and development of drug resistance in an indigent population. AIDS.

[19] Ncaca LN, Kranzer K, Orrell C (2011). Treatment interruption and variation in tablet taking behaviour result in viral failure: a case-control study from Cape Town, South Africa. PLoS ONE.

[20] Orrell C, Mauff K, Bangsberg D, Maartens G, Wood R (2015). A randomised controlled trial of real-time electronic adherence monitoring with text message dosing reminders in people starting first-line antiretroviral therapy. J Acquir Immune Defic Syndr.

[21] Orrell C, Kaplan R, Wood R, Bekker LG (2011). Virological breakthrough: a risk factor for loss to followup in a large community-based cohort on antiretroviral therapy. AIDS Res Treat.

[22] Orrell C, Harling G, Lawn SD, Kaplan R, McNally M, Bekker LG (2007). Conservation of first-line antiretroviral treatment regimen where therapeutic options are limited. Antivir Ther.

[23] Haberer JE, Kahane J, Kigozi I, Emenyonu N, Hunt P, Martin J (2010). Real-time adherence monitoring for HIV antiretroviral therapy. AIDS Behav.

[24] Sabin LL, Bachman DeSilva M, Gill CJ, Zhong L, Vian T, Wubin X (2015). Improving adherence to antiretroviral therapy with triggered real time text message reminders: the China through technology study (CATS). J Acquir Immune Defic Syndr.

[25] National Insititute on Alcohol Abuse and Alcoholism. CAGE questionnaire. http://pubs.niaaa.nih.gov/publications/inscage.htm. 2002.

[26] Herrmann C (1997). International experiences with the Hospital Anxiety and Depression Scale—a review of validation data and clinical results. J Psychosom Res.

[27] Nglazi MD, Kaplan R, Wood R, Bekker LG, Lawn SD (2010). Identification of losses to follow-up in a community-based antiretroviral therapy clinic in South Africa using a computerized pharmacy tracking system. BMC Infect Dis.

[28] Orrell C, Dipenaar R, Killa N, Tassie JM, Harries AD, Wood R (2013). Simplifying HIV cohort monitoring–pharmacy stock records minimize resources necessary to determine retention in care. J Acquir Immune Defic Syndr.

[29] van Leth F, Phanuphak P, Ruxrungtham K, Baraldi E, Miller S, Gazzard B (2004). Comparison of first-line antiretroviral therapy with regimens including nevirapine, efavirenz, or both drugs, plus stavudine and lamivudine: a randomised open-label trial, the 2NN Study. Lancet.

[30] Puls R, Amin J, Losso M, Phanuphak P, Nwizu C, Orrell C (2014). Efficacy of 400 mg efavirenz versus standard 600 mg dose in HIV-infected, antiretroviral-naive adults (ENCORE1): a randomised, double-blind, placebo-controlled, non-inferiority trial. Lancet.

[31] van Zyl GU, Claassen M, Engelbrecht S, Laten JD, Cotton MF, Theron GB (2008). Zidovudine with nevirapine for the prevention of HIV mother-to-child transmission reduces nevirapine resistance in mothers from the Western Cape, South Africa. J Med Virol.

[32] Wensing AM, Calvez V, Gunthard HF, Johnson VA, Paredes R, Pillay D (2014). 2014 Update of the drug resistance mutations in HIV-1. Top Antivir Med.

[33] Wu P, Johnson BA, Nachega JB, Wu B, Ordonez CE, Hare AQ (2014). The combination of pill count and self-reported adherence is a strong predictor of first-line ART failure for adults in South Africa. Curr HIV Res.

[34] Lester RT, Ritvo P, Mills EJ, Kariri A, Karanja S, Chung MH (2010). Effects of a mobile phone short message service on antiretroviral treatment adherence in Kenya (WelTel Kenya1): a randomised trial. Lancet.

[35] Hardy H, Kumar V, Doros G, Farmer E, Drainoni ML, Rybin D, Myung D, Jackson J, Backman E, Stanic A, Skolnik PR (2011). Randomized controlled trial of a personalized cellular phone reminder system to enhance adherence to antiretroviral therapy. AIDS Patient Care STDS.

[36] Da Costa TM, Barbosa BJP, E Costa DAG, Sigulem D, De Fátima Marin H, Filho AC (2012). Results of a randomized controlled trial to assess the effects of a mobile SMS-based intervention on treatment adherence in HIV/AIDS-infected Brazilian women and impressions and satisfaction with respect to incoming messages. Int J Med Inform.

[37] Maduka O, Tobin-West CI (2013). Adherence counseling and reminder text messages improve uptake of antiretroviral therapy in a tertiary hospital in Nigeria. Niger J Clin Pract.

[38] Petersen ML, LeDell E, Schwab J, Sarovar V, Gross R, Reynolds N (2015). Super learner analysis of electronic adherence data improves viral prediction and may provide strategies for selective HIV RNA monitoring. J Acquir Immune Defic Syndr.

[39] Haberer JE, Musinguzi N, Boum Y, Siedner MJ, Mocello AR, Hunt PW (2015). Duration of antiretroviral therapy adherence interruption is associated with risk of virologic rebound as determined by real-time adherence monitoring in rural Uganda. J Acquir Immune Defic Syndr.

[40] Phillips AN, Cambiano V, Nakagawa F, Bansi-Matharu L, Sow PS, Ehrenkranz P (2016). Cost effectiveness of potential ART adherence monitoring interventions in Sub-Saharan Africa. PLoS ONE.

[41] Nglazi MD, Kaplan R, Orrell C, Myer L, Wood R, Bekker LG (2013). Increasing transfers-out from an antiretroviral treatment service in South Africa: patient characteristics and rates of virological non-suppression. PLoS ONE.

[42] Nglazi MD, Lawn SD, Kaplan R, Kranzer K, Orrell C, Wood R (2011). Changes in programmatic outcomes during 7 years of scale-up at a community-based antiretroviral treatment service in South Africa. J Acquir Immune Defic Syndr.

