# Supplementary material for: Comparison of six methods to estimate adherence in an ART-naïve cohort in a resource-poor setting: which best predicts virological and resistance outcomes?
Source: AIDS Res Ther. 2017 Apr 4;14:20. doi: 10.1186/s12981-017-0138-y (PMC5379739; doi:10.1186/s12981-017-0138-y)
Supplement: Supplementary file 1 — Additional file 1. Supplementary tables. [file 12981_2017_138_MOESM1_ESM.docx]

**Supplementary tables:**

Supplementary table 1: Visit and procedure table differentiated by SA National ART guidelines procedures (Standard of Care / SoC visit) and TAP study procedures (Study visit).

| Weeks on ART | Week -4 | Week 0 | Week 4 | Week 8 | Week 16 | Week 24 | Week 32 | Week 40 | Week 48 |
| --- | --- | --- | --- | --- | --- | --- | --- | --- | --- |
| SA National ART Guidelines (SoC) | | | | | | | | | |
| Clinical exam | X | X | X | X | X | X | X | X | X |
| Drug dispensing | X | X | X | X | X | X | X | X | X |
| Clinic-based Pill count | X | X | X | X | X | X | X | X | X |
| CD4 cell count | X |  |  |  |  |  |  |  | X |
| Viral load |  |  |  |  | X |  |  |  | X |
| TAP study visits and study-specific procedures | | | | | | | | | |
| Informed consent | X |  |  |  |  |  |  |  |  |
| Randomisation^#^ |  | X |  |  |  |  |  |  |  |
| 3-day self-report |  |  |  |  | X |  | X |  | X |
| EFV concentration |  |  |  |  | X |  | X |  | X |
| EAMD data |  |  |  |  | X |  | X |  | X |
| Storage* |  |  |  |  |  |  |  |  |  |
| CD4 cell count |  |  |  |  | X |  |  |  |  |
| Viral load | X |  |  |  |  |  |  |  |  |

^#^ into parent study

* buffy layer for cytochrome 2B6 genotyping and plasma for viral genotyping.

Supplementary table 2: Full multivariable logistic regression models predicting virological outcomes at week 16 and 48 using EAMD and PR-average adherence measure.

2a. Full model predicting virologic outcomes at week 48, with EAMD as the adherence measure (n=179)

| Week 48 failure | Odds Ratio | Std. Error | p-value | 95% confidence intervals |
| --- | --- | --- | --- | --- |
| Week 48 EAMD | 0.97 | 0.01 | 0.001 | 0.95-0.99 |
| Age (years) | 0.97 | 0.03 | 0.221 | 0.92-1.02 |
| Baseline CD4 | 0.99 | 0.00 | 0.070 | 0.99-1.00 |
| Log baseline VL | 1.06 | 0,53 | 0.901 | 0.40-2.82 |

2b. Full model predicting virologic outcomes at week 48, with PR-average as the adherence measure (n=177)

| Week 48 failure | Odds Ratio | Std. Error | p-value | 95% confidence intervals |
| --- | --- | --- | --- | --- |
| Week 48 PR-average | 0.94 | 0.12 | <0.01 | 0.92-0.97 |
| Age | 0.97 | 0.03 | 0.366 | 0.92-1.03 |
| Baseline CD4 | 0.99 | 0.00 | 0.013 | 0.99-1.00 |
| Log baseline VL | 0.77 | 0.42 | 0.635 | 0.27-2.23 |

2c. Full model predicting virologic outcomes at week 16, with EAMD as the adherence measure (n=159)

| Week 16 failure | Odds Ratio | Std. Error | p-value | 95% confidence intervals |
| --- | --- | --- | --- | --- |
| Week 16 EAMD | 0.99 | 0.01 | 0.459 | 0.97-1.01 |
| Age | 0.87 | 0.05 | 0.024 | 0.77-0.98 |
| Baseline CD4 | 0.99 | 0.00 | 0.007 | 0.98-1.00 |
| Log baseline VL | 3.99 | 3.44 | 0.109 | 0.73-21.6 |

2d. Full model predicting virologic outcomes at week 16, with PR-average as the adherence measure (n=157)

| Week 16 failure | Odds Ratio | Std. Error | p-value | 95% confidence intervals |
| --- | --- | --- | --- | --- |
| Week 16 PR-average | 0.91 | 0.03 | <0.01 | 0.85-0.97 |
| Age | 0.88 | 0.05 | 0.029 | 0.78-0.98 |
| Baseline CD4 | 0.99 | 0.00 | 0.014 | 0.98-1.00 |
| Log baseline VL | 9.07 | 10.8 | 0.063 | 0.89-92.5 |

Supplementary table 3:

Key categorical models at week 48 for both virological failure and resistance. EAMD data is linearly associated with failure, whereas the relationship of EFV concertation with failure is not linear.

1. Week 48, virological failure:

| Adherence measure | Univariate model | | | Multivariable model | |
| --- | --- | --- | --- | --- | --- |
| **EAMD** | **OR (95%CI)** | | **p-value** | **aOR (95%CI)** | **p-value** |
| Virological failure (>40 copies/ml) at week 48: | | | | | |
| EAMD: quartile 2 vs 1 | 0.32 (0.11-0.94) | 0.039 | | 0.41 (0.13-1.27) | 0.124 |
| EAMD: quartile 3 vs 1 | 0.18 (0.05-0.62) | 0.007 | | 0.21 (0.06-0.74) | 0.015 |
| EAMD: quartile 4 vs 1 | 0.14 (0.04-0.56) | 0.005 | | 0.18 (0.05-0.71) | 0.015 |

| Adherence measure | Univariate model | | Multivariable model | |
| --- | --- | --- | --- | --- |
| **EFV concentration** | **OR (95%CI)** | **p-value** | **aOR (95%CI)** | **p-value** |
| Virological failure (>40 copies/ml) at week 48: | | | | |
| EFV: quartile 2 vs 1 | 0.43 (0.10-1.88) | 0.265 | 0.37 (0.08-1.71) | 0.203 |
| EFV: quartile 3 vs 1 | 0.97 (0.29-3.31) | 0.963 | 0.96 (0.27-3.39) | 0.947 |
| EFV: quartile 4 vs 1 | 0.60 (0.16-2.29) | 0.452 | 0.65 (.016-2.59) | 0.538 |

1. Week 48, resistance:

| Adherence measure | Univariate model | | | Multivariable model | |
| --- | --- | --- | --- | --- | --- |
| **EAMD** | **OR (95%CI)** | **p-value** | | **aOR (95%CI)** | **p-value** |
| Virological failure (>40 copies/ml) at week 48: | | | | | |
| EAMD: combined quartiles 2, 3 & 4 vs quartile 1. | 0.03 (0.00-0.24) | | 0.001 | 0.05 (0.00-0.44) | 0.007 |

| Adherence measure | Univariate model | | Multivariable model | |
| --- | --- | --- | --- | --- |
| **EFV concentration** | **OR (95%CI)** | **p-value** | **aOR (95%CI)** | **p-value** |
| Virological failure (>40 copies/ml) at week 48**:** | | | | |
| EFV: quartiles3&4 vs quartiles 1&2. | 0.19 (0.02-1.64) | 0.131 | 0.40 (0.13-1.23) | 0.109 |
